# UV Curing of Biobased Electrically Conductive Coatings with Covalent Adaptable Network Properties

**DOI:** 10.3390/polym18172058

**Published:** 2026-08-25

**Authors:** Serena Greppi, Alberto Cellai, Rafael Turra Alarcon, Alejandro Cortés Fernández, Alberto Jiménez Suárez, Marco Sangermano

**Affiliations:** 1 Dipartimento di Scienza Applicata e Tecnologia, Politecnico di Torino, C.so Duca degli Abruzzi 24, 10129 Torino, Italyalberto.cellai@polito.it (A.C.); 2 Instituto de Química de São Carlos, Universidade de São Paulo-USP, San Carlos 13566-590, SP, Brazil; rafael.alarcon@ufms.br (R.T.A.); alberto.jimenez.suarez@urjc.es (A.J.S.); 3 Universidad Rey Juan Carlos, Via Tulipán, 28933 Móstoles, Madrid, Spain; alejandro.cortes@urjc.es

**Keywords:** biobased, photocurable, vitrimer, coatings, healing, CANs

## Abstract

The development of sustainable coatings that combine reprocessability with active functionalities remains a central challenge for the composites sector. In this work, a healable, electrically conductive coating was formulated using epoxidized castor oil (ECO) as a bio-based matrix, dibutyl phosphate (DBP) as a transesterification catalyst, and short recycled carbon fibres (RCFs, 2 mm in length) as a conductive filler at loadings of 10 and 20 phr. Formulations were UV-cured via cationic photopolymerization and characterized across the full liquid-to-solid processing chain. FT-IR and photo-DSC showed that increasing RCF content progressively reduced curing rate and conversion, an effect attributed to light scattering/absorption by the fibres and restricted chain mobility, although gel content remained above 98% in all cases. DMTA showed that RCF did significantly affect the glass transition temperature but markedly increased the rubbery storage modulus and apparent crosslink density, consistent with a physical reinforcement mechanism. Stress relaxation tests confirmed the dynamic bond exchange behaviour in all formulations, with the apparent activation energy decreasing from 112 kJ/mol for the neat resin to 33–34 kJ/mol upon RCF incorporation. This significant reduction suggests that the presence of RCF facilitates the bond-exchange process, potentially through interfacial interactions between the polymer network and the fibre surface. However, the specific molecular mechanism responsible for this effect cannot be established from the present data. Electrical conductivity peaked at 10 phr RCF (3.6 × 10^−3^ S/m), enabling measurable Joule heating, while the 20 phr formulation showed reduced conductivity linked to voids and lower conversion. Thermally triggered healing at 120 °C for 6 h restored mechanical integrity, which is higher than reference values, demonstrating the coating’s capacity for repeated repair through its dynamic covalent network.

## 1. Introduction

The shift toward a circular, sustainable economy is no longer optional for materials science; it is a structural requirement [1,2,3,4,5,6,7]. Thermosetting resins based on bisphenol A (BPA) have long dominated the composites and coatings sector due to their mechanical performance, thermal stability, and chemical resistance [8]. Yet the permanence of their covalent crosslinks, the very feature that makes them so robust, also makes them impossible to melt, reshape, or recycle, and this has become one of the sector’s most pressing environmental liabilities [9]. At the same time, the field is being pushed toward polymers that do more than provide structural support: smart materials capable of sensing, actuating, or conducting heat and electricity are increasingly in demand across applications [10,11,12,13,14,15].

Turning an inherently insulating matrix into an electrically active one is central to uses such as electromagnetic shielding and electro-thermal actuation [16,17]. Ionic conduction is sufficient for some applications, polymer electrolytes for batteries, for instance, but efficient resistive Joule heating requires a genuine electronic percolation network, which in practice means dispersing conductive fillers throughout the matrix [18,19]. Carbon fibres are particularly attractive fillers given their combination of electrical conductivity and mechanical reinforcement [20]. Their use also intersects with a separate, unresolved problem: the composites industry generates enormous volumes of prepreg scrap, where up to 75% of material can be discarded before end use, alongside components reaching end of life. Mechanically recycling this waste into short, recycled carbon fibres (RCFs) offers a far less energy-intensive route than pyrolysis or solvolysis, while simultaneously supplying the conductive filler the matrix needs [21].

The present work builds directly on this premise, drawing on recent findings [22,23], which showed that mechanically recycled carbon fibres, sieved to an optimal length of 2.0 mm and dispersed in a bio-based epoxy matrix, can reach electrical conductivities as high as 11 S/m due to their high aspect ratio, while also enabling substantial Joule heating at low applied voltages.

On the matrix side, green chemistry has increasingly focused on vegetable oils as renewable alternatives to petrochemical monomers [24,25,26,27]. Among these, castor oil is particularly attractive because of the naturally occurring hydroxyl groups and carbon–carbon double bonds present in its ricinoleic acid backbone [28,29]. Epoxidation of castor oil produces epoxidized castor oil (ECO), a highly reactive bio-based precursor suitable for polymer synthesis. In this work, ECO is crosslinked through UV-induced cationic photopolymerization [28]. Unlike conventional thermal curing, which requires energy-intensive heating, UV curing is rapid, solvent-free, and can be carried out at room temperature. The process is initiated by a sulfonium salt photoinitiator, which promotes an epoxide ring-opening reaction [28,30]. However, a conventionally cured ECO network would remain infusible and non-reprocessable, sharing the same limitations as BPA-based thermosets [24,26,27]. To overcome this issue, the material was designed as a vitrimer, a covalently adaptable network (CAN) capable of rearranging its macromolecular topology at elevated temperatures while maintaining a constant crosslink density [28,29,31,32,33]. This dynamic behaviour is enabled by associative transesterification reactions, which are facilitated by the ester groups naturally present in the triglyceride backbone and the hydroxyl groups both present in the monomer structure as well as generated during epoxide ring-opening [1,8,34]. Since transesterification has a relatively high activation energy, dibutyl phosphate (DP) was incorporated as an organic catalyst to reduce the activation barrier, allowing the network to undergo viscoelastic flow with practical stress-relaxation times at temperatures above approximately 70 °C. Combining this dynamic matrix (ECO + DP) with a percolating RCF network gives rise to a distinctive property: the possibility of on-demand (remotely and at selected locations), electrically triggered healing. Applying a potential difference drives a current through the carbon fibres, generating volumetric heat by the Joule effect [21]. Once the local temperature exceeds the CAN topological transition, DP catalysis activates, the resin softens and flows into cracks or scratches, and new covalent bonds re-form to heal the damage. Crucially, this process not only restores mechanical integrity but also reconnects severed carbon fibres, recovering the original electrical conductivity of the system [21].

Therefore, this work sets out to develop, formulate, and fully characterize a reparable dynamic coating based on ECO, DP, and RCF. A series of liquid formulations was prepared, keeping photoinitiator and catalyst content fixed while systematically varying the concentration of RCF. Photo-DSC (p-DSC) and FT-IR analysis were used to evaluate the curing process. Cured samples were then characterized by dynamic mechanical thermal analysis (DMTA) to determine storage modulus and glass transition temperature, while stress relaxation tests on a rheometer served as proof of dynamic behaviour and catalytic efficiency. Thermo-electrical characterization was performed to assess if the generated conductive percolation network was able to induce Joule heating to be exploited for the dynamic covalent bond activation. Finally, micro-tensile testing was used to quantify the efficiency of thermally triggered healing.

## 2. Materials and Methods

### 2.1. Materials

**Figure 1:**
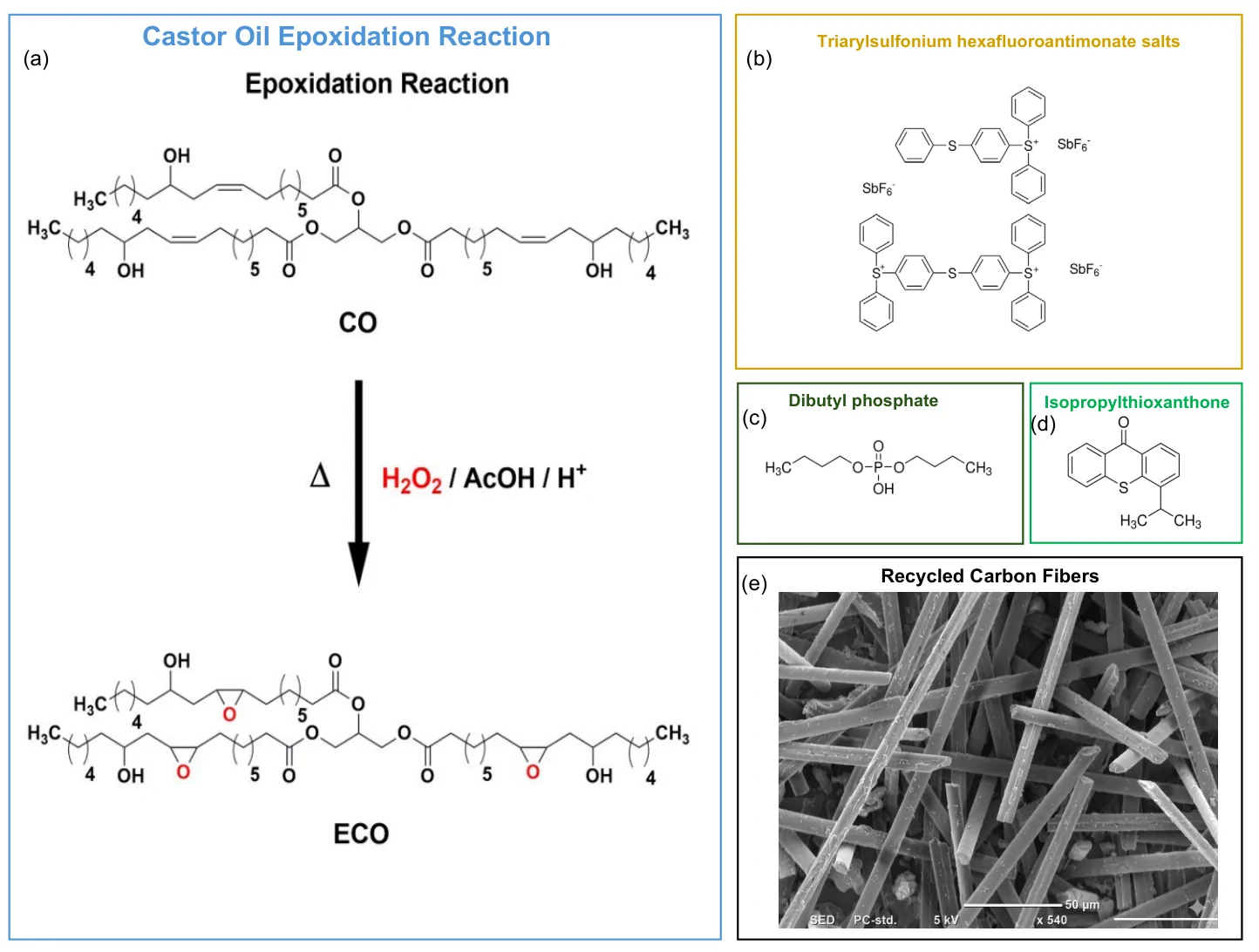
Castor oil epoxidation scheme: (**a**), Triarylsulfonium hexafluoroantimonate salts (**b**), dibutyl phosphate (**c**), Isopropylthioxanthone (**d**) and recycled carbon fibres micrographs (**e**).

Epoxidized castor oil (ECO) was achieved by epoxidizing castor oil. The chemical structure of ECO and the epoxidation reaction are reported in Figure 1a. The castor oil was epoxidized following a procedure described in the literature [35,36]. Castor oil (CO) was purchased from Mundo dos Óleos (Brasília, Brazil).

The cationic photoinitiator, Triarylsulfonium hexafluoroantimonate salts (Figure 1b), dibutyl phosphate (Figure 1c), and Isopropylthioxanthone (ITX, Figure 1d) were purchased from Sigma-Aldrich. All substances were used as received.

Recycled carbon fibres (RCFs) were recovered from dismissed expired prepreg scrap from the aerospace industry by mechanical milling, performing 10 milling cycles at 3000 rpm with a sieve size of 2 mm using a Pulverisette 19 equipment from Fritsch [21]. SEM images of fillers are visible in Figure 1e.

### 2.2. Formulations Preparation

As visible from Table 1, a total of 3 formulations were prepared, including the pristine formulation with no filler addition and formulations with 10 and 20 phr (per hundred resin) of RCF.

In each formulation, 2 phr of cationic photoinitiator, Triarylsulfonium hexafluoroantimonate salts, 2 phr of photosensitizer, Isopropylthioxanthone (ITX), and 15 phr of transesterification catalyst, dibutyl phosphate, were added to epoxidized castor oil (ECO). Afterward, a THINKYMIXER ARE-250 planetary mixer was used to homogeneously distribute fillers in the monomer and degas the formulations, according to the following programme: 1 min mixing and then 1 min degassing at 1600 rpm, 1 min mixing and then 1 min degassing at 1800 rpm, and finally 2 min mixing at 2000 rpm.

### 2.3. Characterization of Liquid Formulations

#### 2.3.1. Curing Characterization

Fourier Transform Infrared Spectroscopy (FT-IR) was employed to monitor the crosslinking process using a Thermo Scientific Nicolet iS50 spectrometer (Thermo Fisher Scientific, Norwalk, CT, USA). Liquid formulations were applied onto a SiC substrate, forming a 12 μm thick layer. The films were then irradiated with a DYMAX ECE 5000 Flood lamp under curing conditions corresponding to an irradiance of 177 mW/cm^2^, achieved by positioning the samples at 14 cm from the lamp source. Spectra were collected at a resolution of 4 cm^−1^, and data analysis was performed using OMNIC 9.2 software (Thermo Fisher Scientific). The photocuring process was followed by observing the reduction in the oxirane ring absorption band at 825–840 cm^−1^, while the peak at 1727 cm^−1^, attributed to the carboxylic group and considered stable during the reaction, was used as an internal reference. Monomer conversion as a function of exposure time was calculated according to Equation (1):

(1) $$Conversion\;\left(\%\right)=\frac{{\left(\frac{A_{fun}}{A_{ref}}\right)}_{t=0}-{\left(\frac{A_{fun}}{A_{ref}}\right)}_{t}}{{\left(\frac{A_{fun}}{A_{ref}}\right)}_{t=0}}\cdot 100$$

where A_fun_ is the area of the functional group, in this case, the acrylate group at 810 cm^−1^, while A_ref_ is the area of the reference group at 1727 cm^−1^ [37,38,39]. The spectra were collected after different exposure times.

Photodifferential scanning calorimetry (photo-DSC) was also performed on the same formulations to confirm the results obtained with FT-IR. The analysis was performed with the Metler TOLEDO DSC-1 instrument equipped with Gas Controller GC100. This test has been used to evaluate the rate and the heat released during the crosslinking process. For each test, 5–15 mg of liquid resin was placed in an aluminum crucible with a volume of 40 μL, while an empty crucible was used as a reference. The analysis was conducted at a constant temperature (100 °C) in a N_2_-controlled atmosphere (40 mL/min) and a Hamamatsu LC8 mercury lamp (Hamamatsu Photonics) at 100% intensity was used for the irradiation of the samples. The samples were subjected to a two-step cycle, each of which was characterized by 2 min with the lamp switched off followed by 5 min of irradiation. The second run was done to create the baseline and to complete the curing process. During data analysis, the second curve was subtracted from the first one to obtain a single curve related to the photopolymerization process of the sample.

It is possible to evaluate the polymerization rate using Equation (2) if the heat released by the samples during the analyses originates solely from the photo-crosslinking process.

(2) $$R_{p}=\frac{1}{\Delta H_{tot}}\cdot {\left(\frac{dH}{dt}\right)}_{T}$$

Here, $R_{p}$ is the polymerization rate [s^−1^], $\Delta H_{tot}$ is the enthalpy released by complete crosslinking of the sample [J/g], calculated using Equation (3), and dH/dt is the heat flow measured under isothermal conditions (25 °C) [J/g·s].

(3) $$\Delta H_{tot}=n\cdot f\cdot H_{ep}\cdot \frac{1}{PM}$$

Here, n is the number of epoxy rings per mole of monomer, f is the fraction of epoxy rings that react to form the polymer network rather than participating in other reactions (in this work, f=1), $H_{ep}$ is the theoretical enthalpy for the opening of one mole of epoxy rings [J] (= −74,000 J/mol [28]), and PM is the molecular weight of the monomer [g/mol] (calculated as approximately 975 g/mol for ECO).

By integrating Equation (2), it is possible to obtain an estimate of the conversion percentage (C), according to Equation (4).

(4) $$C=\frac{1}{\Delta H_{tot}}\cdot \int_{0}^{t}{\left(\frac{dH}{dt}\right)}_{T}$$

For both these analyses, the measurements were performed three times for each sample.

#### 2.3.2. Rheology

Rheological characterization was performed to evaluate the viscosity behaviour of the prepared formulations as a function of fibre content and to assess the quality of fibre dispersion within the two investigated resin matrices. The measurements were carried out using an Anton Paar MCR 302e™ rotational rheometer equipped with a parallel-plate geometry.

Two parallel plates with a diameter of 25 mm were employed, maintaining a 0.5 mm gap between them, at ambient temperature.

For each measurement, enough of the uncured resin formulation was placed between the plates to completely fill the testing gap. The viscosity was then recorded over a shear rate range from 0.01 to 100 s^−1^. The rheological behaviour of each formulation was subsequently represented by plotting viscosity (Pa·s) as a function of shear rate (s^−1^), allowing the influence of fibre loading on the flow properties of the systems to be evaluated.

### 2.4. Characterization of the Cured Samples

#### 2.4.1. Gel Content

The gel content (%gel) was determined by evaluating the mass of the insoluble fraction remaining after solvent extraction. Cured specimens (approximately 200 mg) were immersed in chloroform for 24 h at room temperature to remove any soluble species, including unreacted monomers, oligomers, and non-crosslinked polymer fractions. Following extraction, the samples were first dried under ambient conditions for 24 h and then further dried at 60 °C for 6 h to ensure complete evaporation of residual solvent before weighing.

The gel content was calculated as the ratio between the final dry mass after extraction (W_1_) and the initial mass before extraction (W_0_), according to Equation (5):

(5) $$\%gel=\frac{W_{1}}{W_{0}}\times 100$$

where W_0_ is the initial mass of the cured specimen and W_1_ is the mass of the insoluble residue after solvent extraction and drying. The gel fraction provides an indirect measure of the crosslinking extent and network integrity, with higher values indicating the formation of a more interconnected and solvent-resistant structure. All measurements were performed in triplicate for each formulation, and the reported values correspond to the average of the three determinations.

#### 2.4.2. Thermo-Mechanical Characterizations

Viscoelastic properties were investigated using a Triton Technology instrument model Tritec 2000 in tension mode. The measurements were done using uniaxial stress with a frequency of 1 Hz. The starting temperature of −60 °C was achieved using liquid nitrogen. Then the sample was heated at a rate of 3 °C/min until a rubbery plateau was reached. This kind of analysis was used to determine the glass transition temperature, which corresponds to the peak of the TanDelta vs. temperature curve. DMTA was also used to calculate the crosslinking density (ν_c_) through Equation (6):

(6) $$\nu_{C}=\frac{E^{\prime }}{3RT}$$

Here, ν_c_ is the number of crosslinks per volume of the crosslinked network; E’ is the storage modulus in the rubbery plateau registered at temperature T = T_g_ + 50 °C, expressed in Kelvin and R is the gas constant. The samples used in this analysis were prepared by casting the formulations in a mold (average dimensions of 0.5 × 3.5 × 13 mm), and then they were cured using the DYMAX ECE Flood lamp (Dymax Europe GmbH) with a light intensity of 150 mW/cm^2^ for 120 s per side.

#### 2.4.3. TGA

Thermogravimetric analysis (TGA) was performed on pristine and 20 phr RCF crosslinked DGEVA using a Mettler-Toledo TGA/DSC 3+ STARe System. Samples with a mass of approximately 10.0 mg were placed in open α-alumina crucibles (200 μL) and heated from 25 °C to 900 °C at a rate of 10 °C/min under a continuous flow of dry air (40.0 mL/min).

#### 2.4.4. Investigation of Dynamic Polymer Network Properties

Stress-relaxation tests were performed on the UV-cured samples using an Anton Paar MCR 302e rheometer. Discs with an average diameter of 25 mm and a thickness of about 1 mm were used. Each specimen was preloaded under a normal force of 1 N for 15 min at the selected temperature to ensure proper contact and thermal equilibration. A constant strain of 3% was then applied, and the decay of the relaxation modulus over time was recorded. Measurements were carried out at 150, 160, 170, and 180 °C.

The relaxation modulus, G(t), was normalized with respect to its initial value, G(t_0_). The characteristic relaxation time of the dynamic polymer network was defined as the time required for the normalized modulus to drop to 1/*e* (≈37%) of its initial value, assuming an exponential decay behaviour, as described by Equation (7) [40]:

(7) $$G\left(t\right)=G_{t0}e^{-\frac{t}{\tau }}$$

where τ is the relaxation time [s]. The activation energy was determined by extrapolating the Arrhenius plot, as per Equation (8):

(8) $$\ln\left(\tau \right)=\ln\left(\tau_{0}\right)+E_{a}\frac{1000}{RT}$$

where *E_a_* is the activation energy [J/mol], R is the universal gas constant [J/mol*K], T is the temperature [K], and τ_0_ is a pre-exponential factor. By plotting ln(τ) vs. 1000/T, it is possible to calculate E_a_ using Equation (9), considering the slope of the curve (*a*).

(9) $$E_{a}=a\times 1000\times R$$

#### 2.4.5. Thermo-Electrical Properties

Conductivity and Joule effect analysis were performed on samples similar to those for DMTA, as discussed in Section 2.4.2, for each formulation.

The electrical conductivity tests were carried out using a Keithley 2410 source-metre. Silver conductive paint was used on two opposite faces of the specimen to minimize contact resistance. Here, the electrical resistance (R) was calculated from the voltage intensity (V-I) slope, considering Ohm’s law, by sweeping the voltage from 0 to 3 V. Then, the electrical conductivity (σ) was calculated from Equation (10), where L is the distance between the electrodes, and A is the cross-sectional area.

(10) $$\sigma =\frac{L}{A\times R}$$

**Figure 2:**
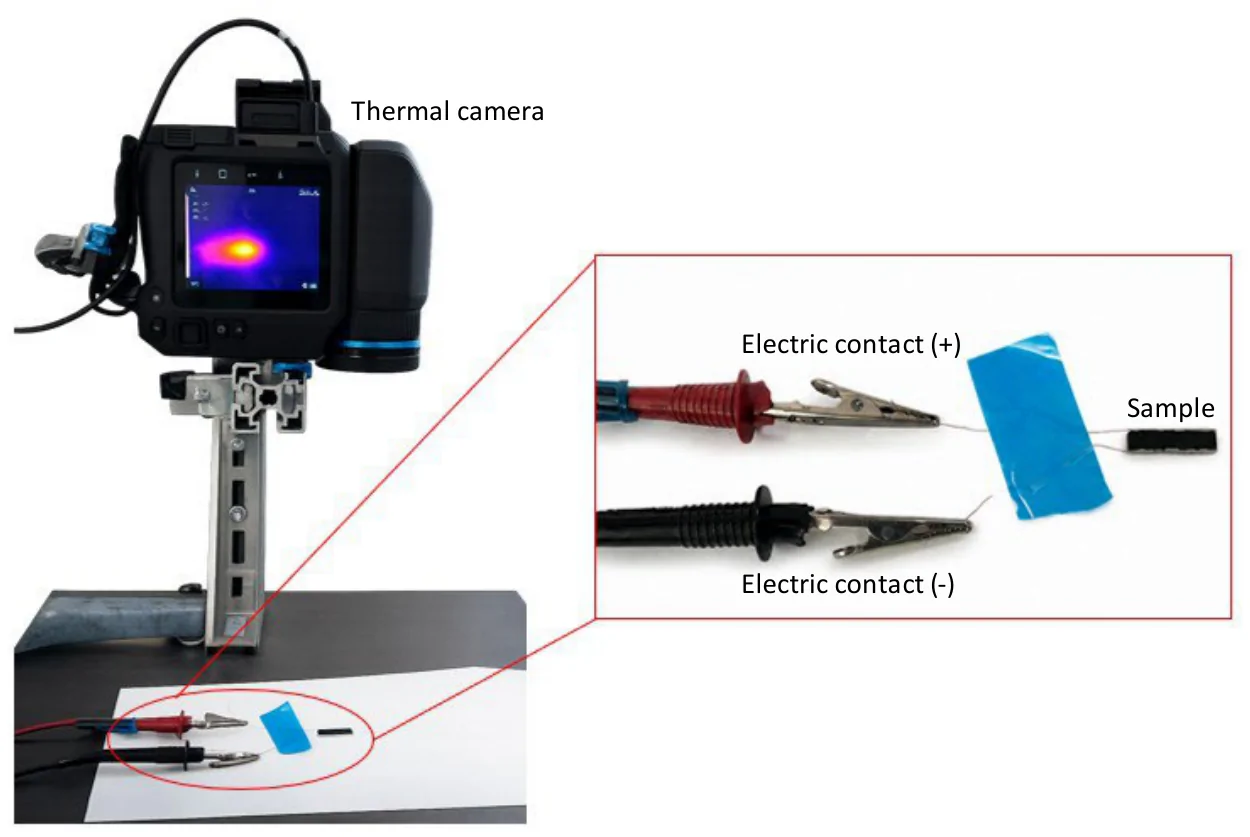
Setup for electrical conductivity and Joule heating measurements.

The Joule heating characterization was carried out using a FLIR T530 thermal camera while applying voltage ranging from 0 V up to a maximum value selected depending on the resistivity of the material using the same source-metre equipment and specimens as in the electrical characterization tests. A picture of the setup is reported in Figure 2. Here, the maximum and average temperatures, T_max_ and T_av_, respectively, were recorded once the temperature was stable over time for each applied voltage.

#### 2.4.6. Micro-Tensile Measurement

Healing efficiency of ECO samples was assessed via mechanical characterization using a Hitachi micro-tensile testing instrument equipped with a 50 N load cell, operating at a crosshead displacement speed of 1 mm/min. The same specimen geometry employed for DMTA was used for these tests.

The samples were partially overlapped under a constant applied pressure of 1.29 × 10^−4^ MPa to ensure proper interfacial contact and then subjected to two different thermal healing treatments in an oven: the first was performed at 70 °C for 30 min, and the second was performed at 120 °C for 6 h. After the healing treatments, tensile measurements were carried out using the same micro-tensile setup mentioned above to evaluate the recovered mechanical strength. The results were finally compared with those obtained from a set of untreated reference specimens.

**Figure 3:**
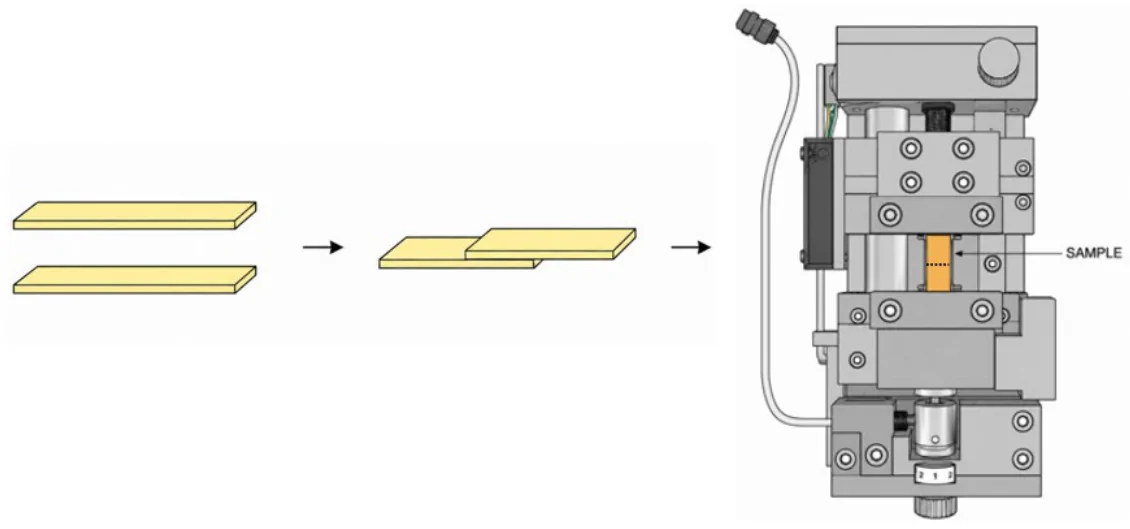
Micro-tensile setup.

A scheme of the setup is visible in Figure 3.

#### 2.4.7. SEM

SEM analyses were conducted to investigate the dispersion of fillers inside the polymeric matrix and adhesion of the formulations with the substrates. More precisely, photos were taken with the Jeol JCM-6000 PLUS for each sample. The analyzed surface was covered with a 10 nm thick platinum layer.

#### 2.4.8. Contact Angle

The contact angles of all cured materials were measured using a Krüss DSA10 tensiometer (Krüss Scientific, Hamburg, Germany) equipped with a digital camera. A droplet of distilled water was placed on the surface of each sample, and ten optical scans were carried out to determine the contact angle between the droplet and the substrate. The measurements were performed in triplicate, following the same experimental procedure.

## 3. Results and Discussion

### 3.1. UV Curing Characterizations

The curing process was evaluated by means of FTIR analysis, following the epoxy group conversion upon UV irradiation.

**Figure 4:**
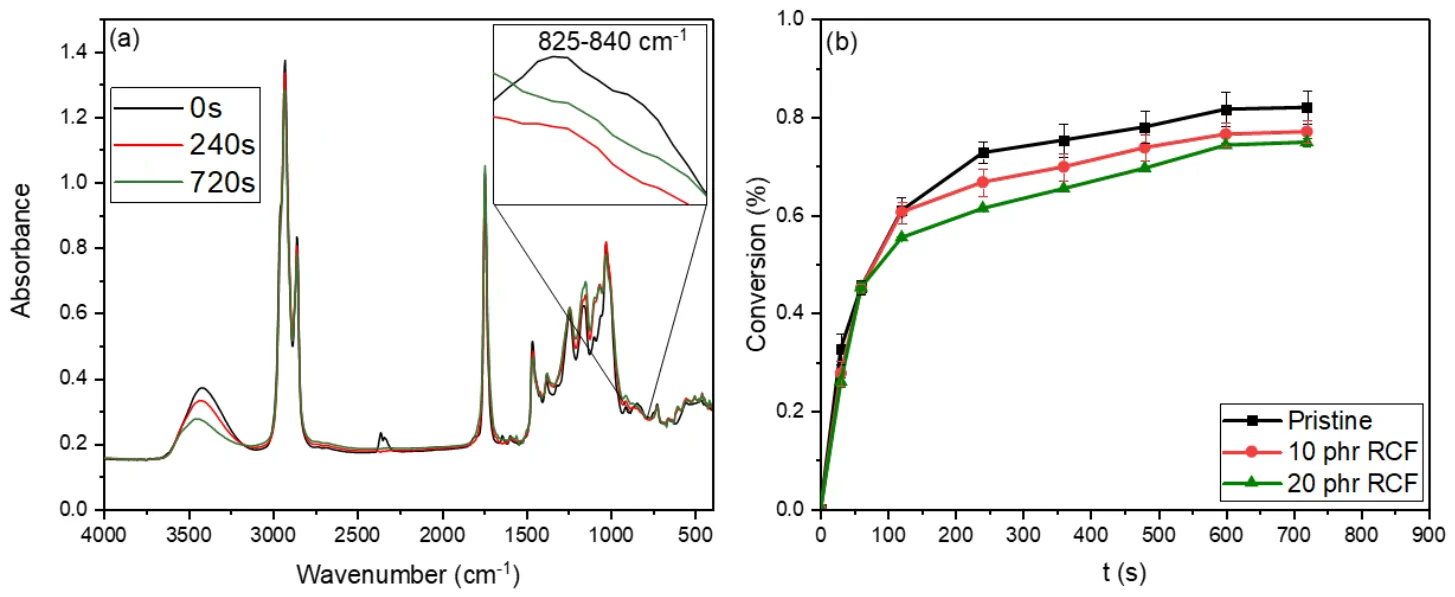
FTIR spectrum of the pristine ECO formulation at different irradiation times (**a**) and conversion curves of all the studied formulations as a function of exposure time (**b**).

Figure 4a shows a representative FTIR spectrum of the pristine ECO formulation.

The evolution of the epoxy conversion during irradiation was monitored by following the absorption band in the 825–840 cm^−1^ region, corresponding to the oxirane ring. The absorption band at 1727 cm^−1^, assigned to the ester carbonyl group, was used as the internal reference (Equation (1)).

Figure 4b reports the conversion curves as a function of UV irradiation time both for pristine and filled formulations. The good reactivity of the pristine biobased epoxy system is evident, reaching almost 80% conversion within 400 s of irradiation. The presence of the RCF slightly affects the conversion curves with a reduction in final epoxy group conversion. This could be attributed to the competitive UV-absorption effect among the photoinitiator and the RCF. Moreover, it could be expected that the filler exerts a physical constraining effect on the developing polymer network, limiting the mobility of the reactive species and consequently reducing the curing efficiency of the matrix.

The curing process was investigated by p-DSC to evaluate the photocuring behaviour and determine the main thermodynamic parameters of the reaction.

**Figure 5:**
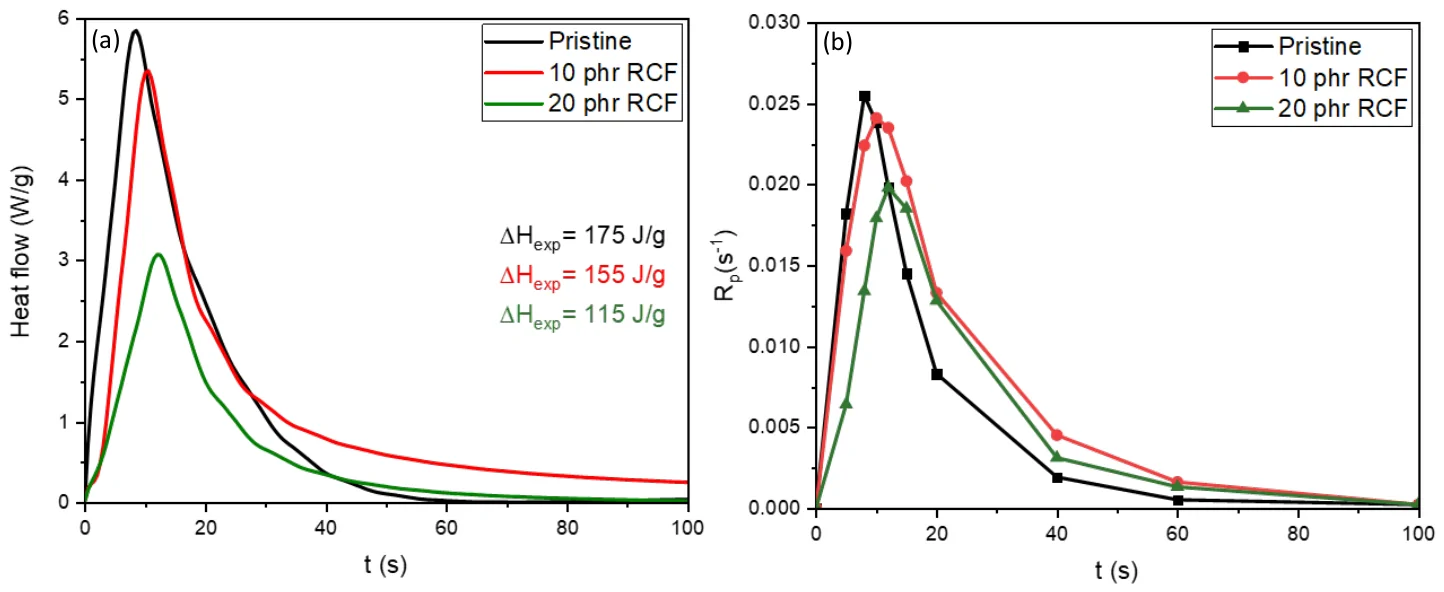
Heat flow (**a**) and rate of polymerization (**b**) for all the studied formulations.

As shown in Figure 5, p-DSC results reveal a progressive decrease in both the measured reaction enthalpy and rate of polymerization R_p_ with increasing RCF content. Specifically, the enthalpy decreases from 175 J g^−1^ for the pristine formulation to 155 J g^−1^ and 115 J g^−1^ for the composites containing 10 phr and 20 phr of RCF, respectively.

This behaviour is consistent with the results obtained from the previous FTIR analyses.

The conversion values obtained from p-DSC and FTIR are in good agreement in terms of their overall trend. In both cases, the degree of conversion decreases as the filler content increases, owing to the same mechanisms discussed above. The slight differences observed in the absolute conversion values can be attributed to the different experimental setups and measurement principles of the two characterization techniques.

Gel content analysis yielded values higher than 98% for all formulations, confirming the formation of a fully interconnected and insoluble network regardless of filler loading.

Table 2 reports conversion values obtained through FTIR and p-DSC, maximum R_p_ and gel content values.

We can conclude that ECO-based formulations showed good reactivity towards the cationic UV-curing process and the addition of the RCF filler did not significantly affect the crosslinking reaction, achieving a well-insoluble crosslinked network.

### 3.2. Rheology

**Figure 6:**
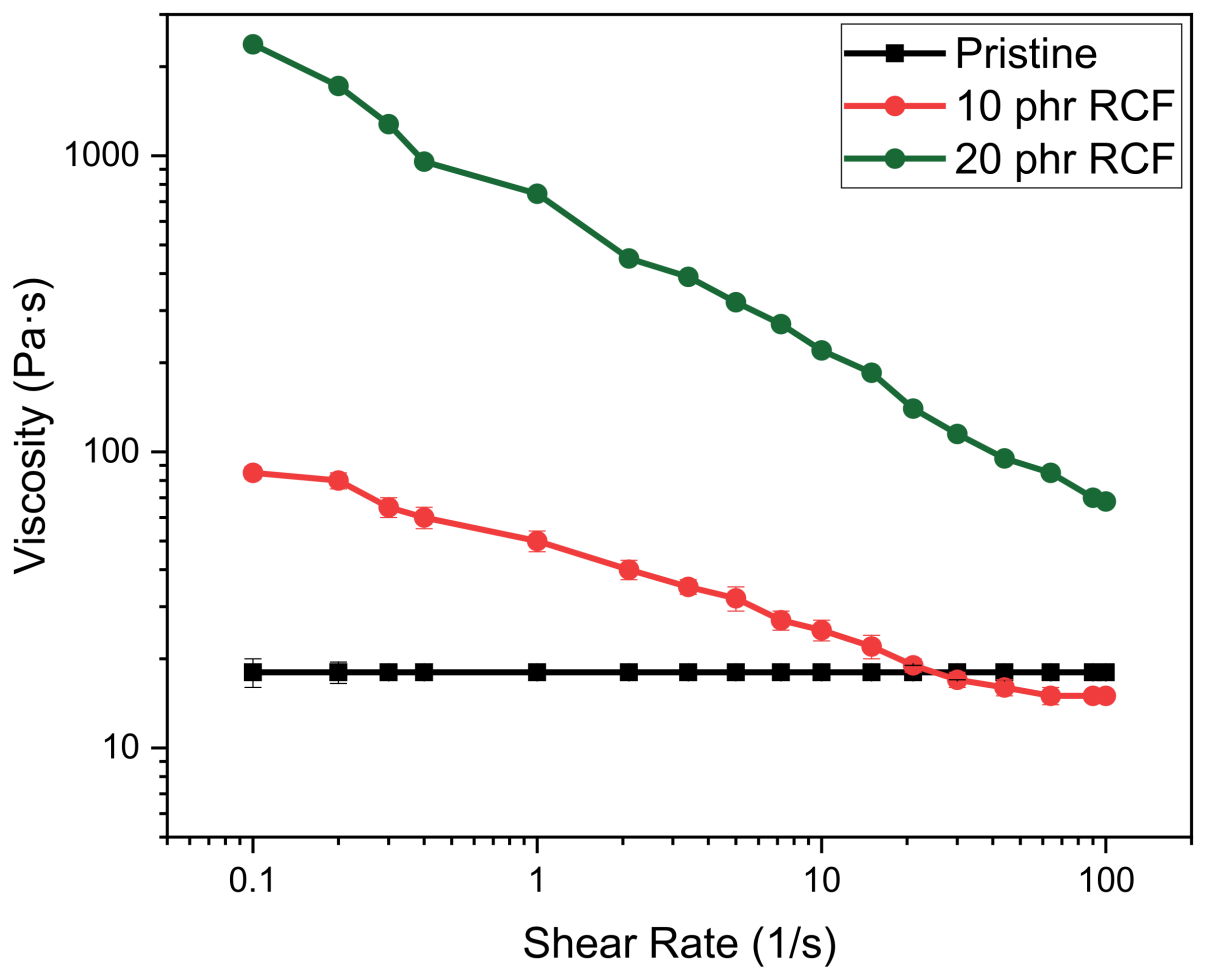
Viscosity as a function of shear rate of the studied formulations.

Rheological measurements were carried out to assess the variation in viscosity as a function of the RCF content, as illustrated in Figure 6. The pristine matrix exhibits a typical Newtonian behaviour across the entire shear rate range, stabilizing at approximately 18 Pa·s. Upon the addition of 10 phr RCF, the system displays a weak shear-thinning behaviour at low shear rates, dropping from an initial value of 85 Pa·s. Notably, at high shear rates, the viscosity of the 10 phr RCF formulation falls near the values of the pristine resin. This phenomenon is ascribed to the strict alignment of the high-aspect-ratio carbon fibres along the flow direction induced by the intense forces, which significantly minimizes the drag and induces interfacial slip, introducing a lubrication effect from the fibres. Additionally, the 20 phr RCF system presents a severe and continuous pseudoplastic behaviour, despite a sharp drop in viscosity due to the progressive breakdown of the robust percolated filler network under dynamic shear; its viscosity remains higher than the pristine matrix across the investigated range due to the high filler volume fraction.

### 3.3. Contact Angle

**Figure 7:**
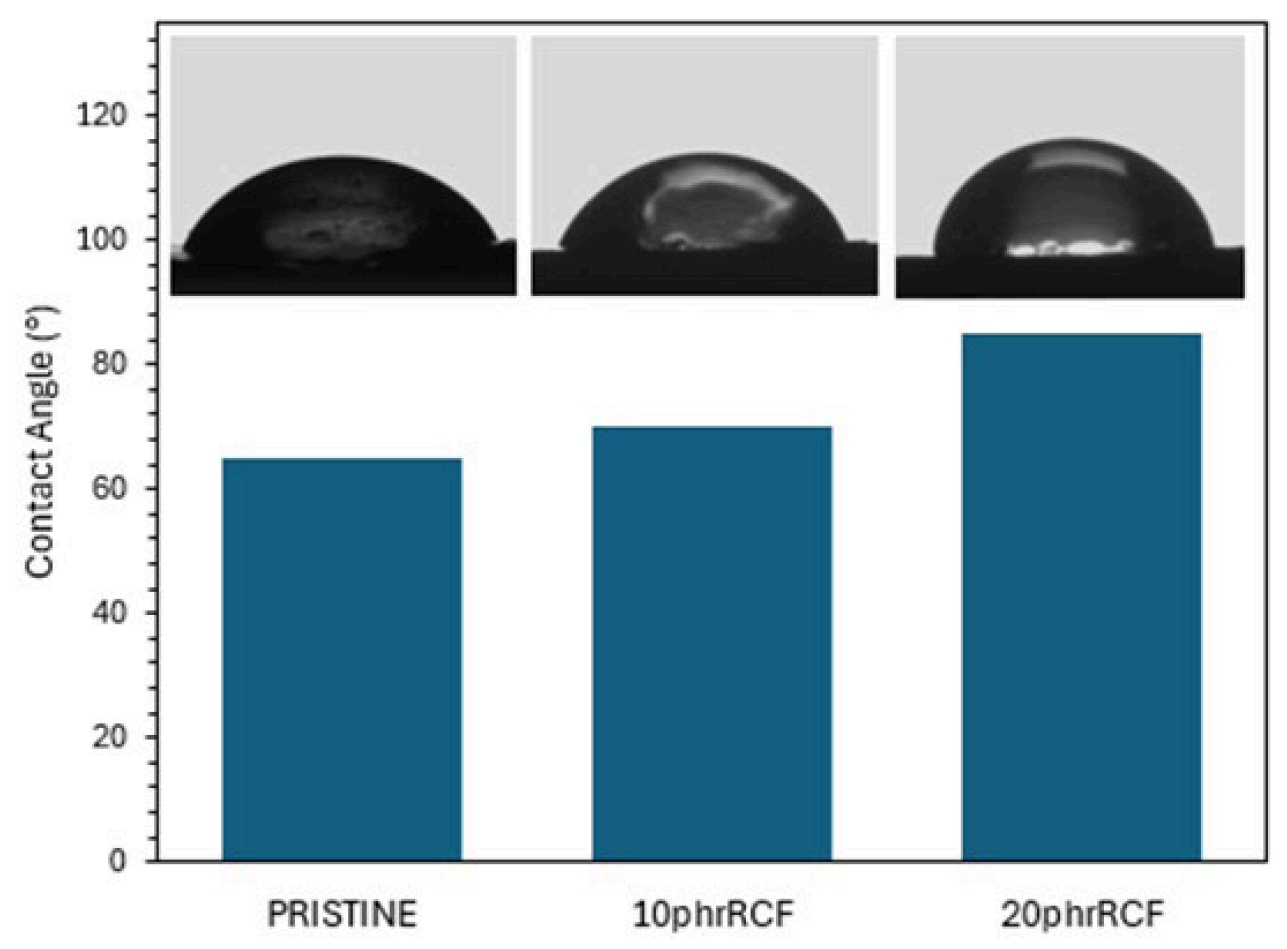
WCA values as a function of RCF content.

Figure 7 reports the water contact angle (WCA) values measured for the different composite formulations. For both resin systems, a consistent trend was observed: the WCA increased progressively with increasing RCF content, rising from approximately 65° for the pristine matrix to roughly 70° and 85° for the 10 phr and 20 phr RCF composites, respectively. This behaviour is driven by a synergetic effect of surface chemistry and topography. Intrinsic carbon fibres exhibit higher hydrophobicity. Consequently, as the filler fraction increases, a larger chemical area of the composite surface is occupied by these low-energy carbon domains. Crucially, the incorporation of RCF increases the micro-roughness and structural heterogeneity of the composite surface. According to the Cassie–Baxter and Wenzel wetting models [41,42,43], this enhanced surface roughness induces pinning of the water triple-line and can trap micro-air pockets beneath the droplet, effectively altering the macroscopic wetting state and leading to a significant reduction in overall surface energy and a concurrent increase in the static contact angle. This increase could be beneficial for several applications, such as anti-icing purposes in cold and humid environments.

### 3.4. Thermo-Mechanical Characteristics

**Figure 8:**
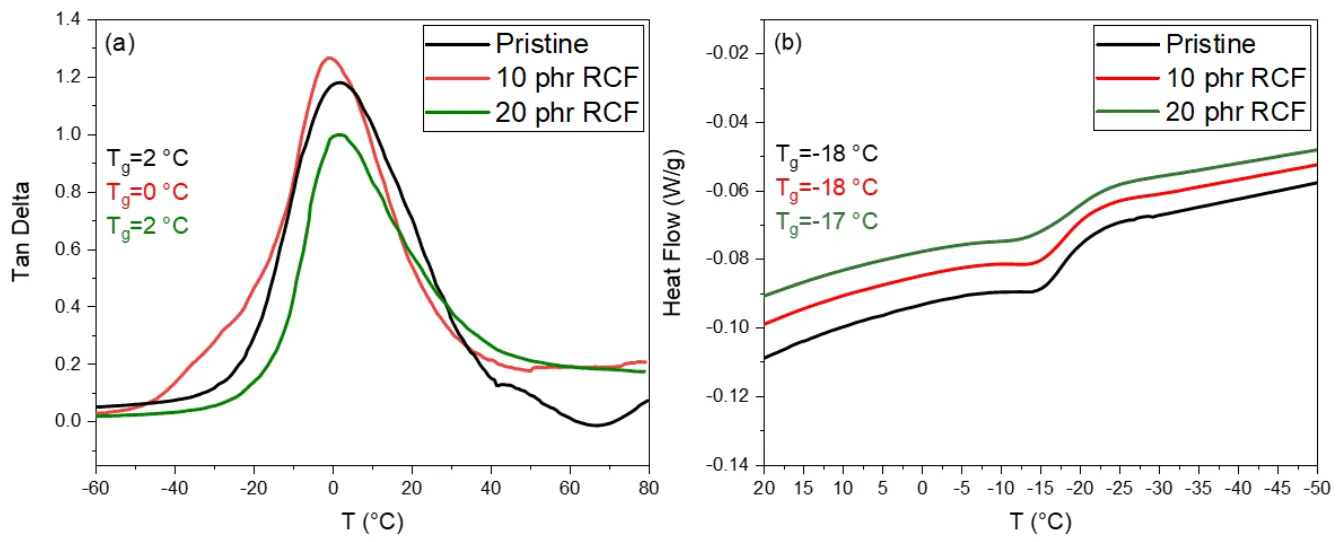
Temperature dependence of TanDelta (**a**) and the DSC thermograms (**b**) of the studied formulations.

The thermal and viscoelastic properties of the UV-cured ECO formulations were evaluated by DMTA and DSC analyses. Figure 8a,b report the temperature dependence of the loss factor (TanDelta) and the DSC thermograms, respectively.

The incorporation of RCF did not produce a significant variation in T_g_. Indeed, DMTA revealed T_g_ values between o °C and 2 °C for the UV-cured formulations.

Similarly, the DSC thermograms show T_g_ values of about −17 and −18 °C.

The ≈20 °C discrepancy between DMTA and DSC T_g_ values is physically consistent, arising from the distinct thermodynamic and kinetic principles of the two analytical techniques. DSC records the change in specific heat capacity under static heating, detecting the onset of localized cooperative segmental mobility. In contrast, DMTA evaluates macroscopic viscoelastic dissipation under a periodic mechanical stress. Because the glass transition is a frequency-dependent kinetic process, higher thermal energy is required for the polymer chains to respond in-phase with the applied oscillating deformation. Furthermore, the analytical extrapolation criteria fundamentally differ: DSC T_g_ is conventionally assigned to the inflection point of the heat flow step, whereas the DMTA T_g_ reported is evaluated at the TanDelta peak. This peak indicates the highest ratio of loss to storage modulus, an event demanding greater free volume that systematically occurs 15–25 °C above the DSC inflection point. Consequently, absolute values from these techniques are not directly comparable; the structural analysis must rely on the relative thermal shifts, which confirm that varying the RCF content does not significantly perturb the crosslinking dynamics of the ECO matrix.

**Figure 9:**
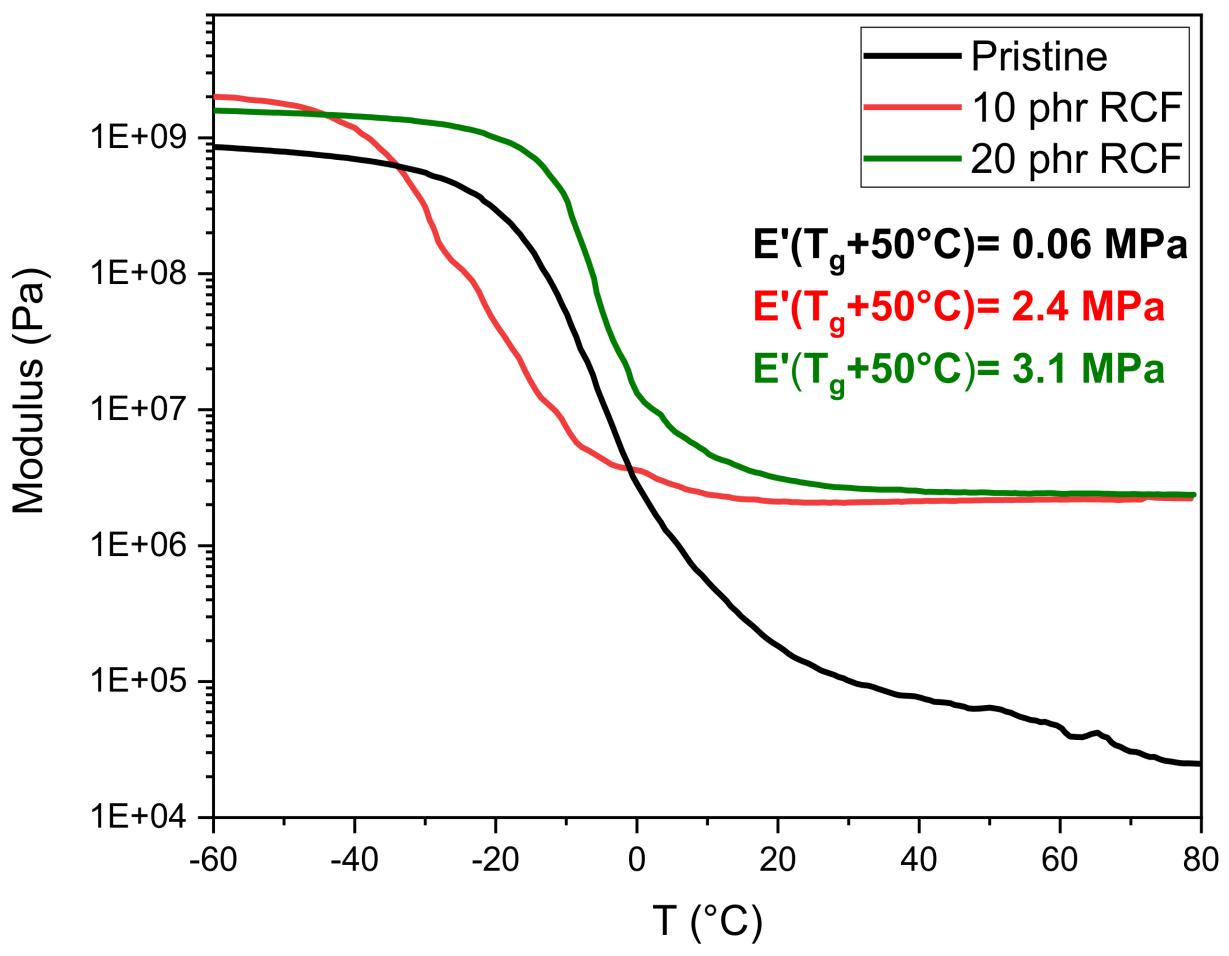
E′ as a function of temperature for all the formulations.

Furthermore, Figure 9 shows the evolution of the storage modulus (E′) as a function of temperature. Although the addition of RCF does not significantly affect T_g_, it markedly increases the modulus in the rubbery region (Tg + 50 °C). The E′ value at Tg + 50 °C rises from 0.06 MPa for the pristine resin to 2.4 MPa and 2.6 MPa for the 10 and 20 phr UV-cured filled formulations, respectively.

In addition, Table 3 reports values of ν_c,app._, showing a dramatic increase upon the introduction of RCF.

The values increased from 7 mol m^−3^ for the neat resin to 298 mol m^−3^ and 320 mol m^−3^ for the 10 and 20 phr composites, respectively.

It is important to emphasize that this substantial increase in ν_c_ does not correspond to an actual change in the chemical crosslinking density of the network. Indeed, DSC and FTIR analyses (Table 2) confirmed a comparable degree of conversion for all formulations. Therefore, the increase in the calculated ν_c_ should be interpreted as an apparent effect arising from physical reinforcement. The rigid carbon fibre network contributes to stress transfer in the rubbery state, while the immobilized polymer chains at the fibre–matrix interface act as additional physical constraints. These results demonstrate that RCFs provide significant reinforcement at more elevated temperatures.

**Figure 10:**
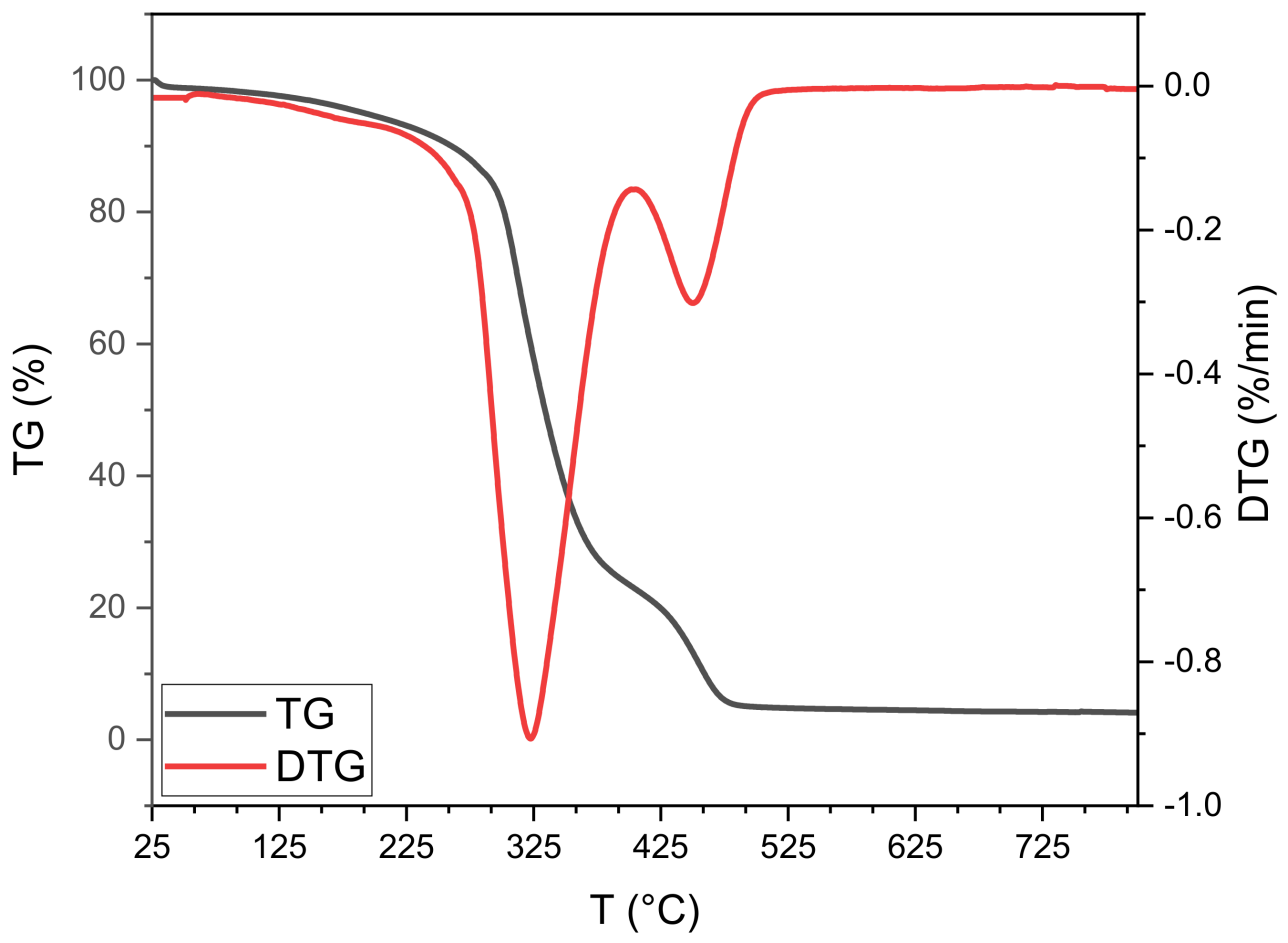
TG and DTG curves of pristine sample.

Thermogravimetric analysis (TGA) was performed on the cured formulations to evaluate their thermal stability. This assessment is particularly important for defining the temperature limits during the stress relaxation process, ensuring that the material is not exposed to temperatures that could induce thermal degradation. The pristine formulation was analyzed and the corresponding TG and DTG curves are reported in Figure 10. The onset degradation temperature, identified by T(5%), was found to be approximately 220 °C, while the maximum degradation rate, corresponding to the DTG peak (T_max_), occurred at 323 °C. Based on these results, particular attention was paid during all subsequent measurements to ensure that the operating temperature remained below the onset degradation temperature T(5%), thus preventing any thermally induced damage to the material.

### 3.5. Covalent Adaptable Network Properties

**Figure 11:**
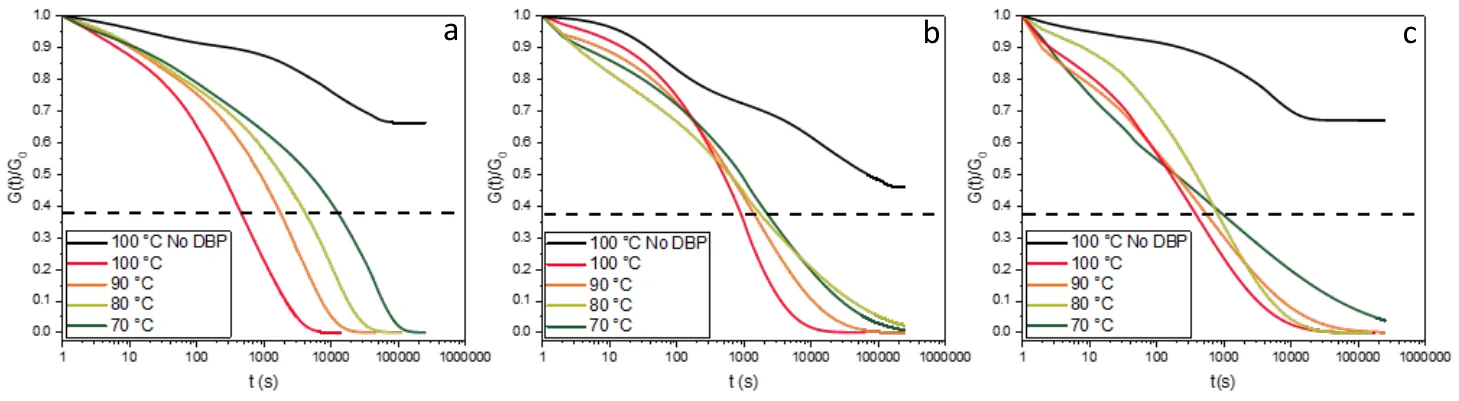
G(t)/G_0_ as a function of time for pristine (**a**), 10 (**b**) and 20 (**c**) phr RCF samples.

Stress-relaxation tests were carried out to demonstrate the dynamic behaviour of the network, consistent with a thermally activated transesterification mechanism, which is chemically expected from the functional groups present in the network. The experiments were conducted within the linear viscoelastic region at temperatures ranging from 70 °C to 100 °C. The dynamic nature of the bonds in ECO is governed by a transesterification reaction. Figure 11 shows, for all three UV-cured systems (pristine, 10 and 20 phr), the normalized relaxation modulus (G(t)/G_0_) as a function of time. As expected, the relaxation rate for all systems increases with increasing temperature. The characteristic relaxation time (τ) is defined as the time required for the modulus to decay to 1/e (≈37%) of its initial value, as indicated by the dashed line [44,45,46]. Moreover, for all formulations, a DBT-catalyst-free reference system was specifically prepared to highlight the different behaviour shown by the black curve in Figure 11. In this case, the material is never able to reach a decay below 1/e (≈37%) of its initial value within the investigated time window, clearly indicating that DBT is the key component enabling bond dynamics in the material by catalyzing the transesterification dynamic covalent reaction.

**Figure 12:**
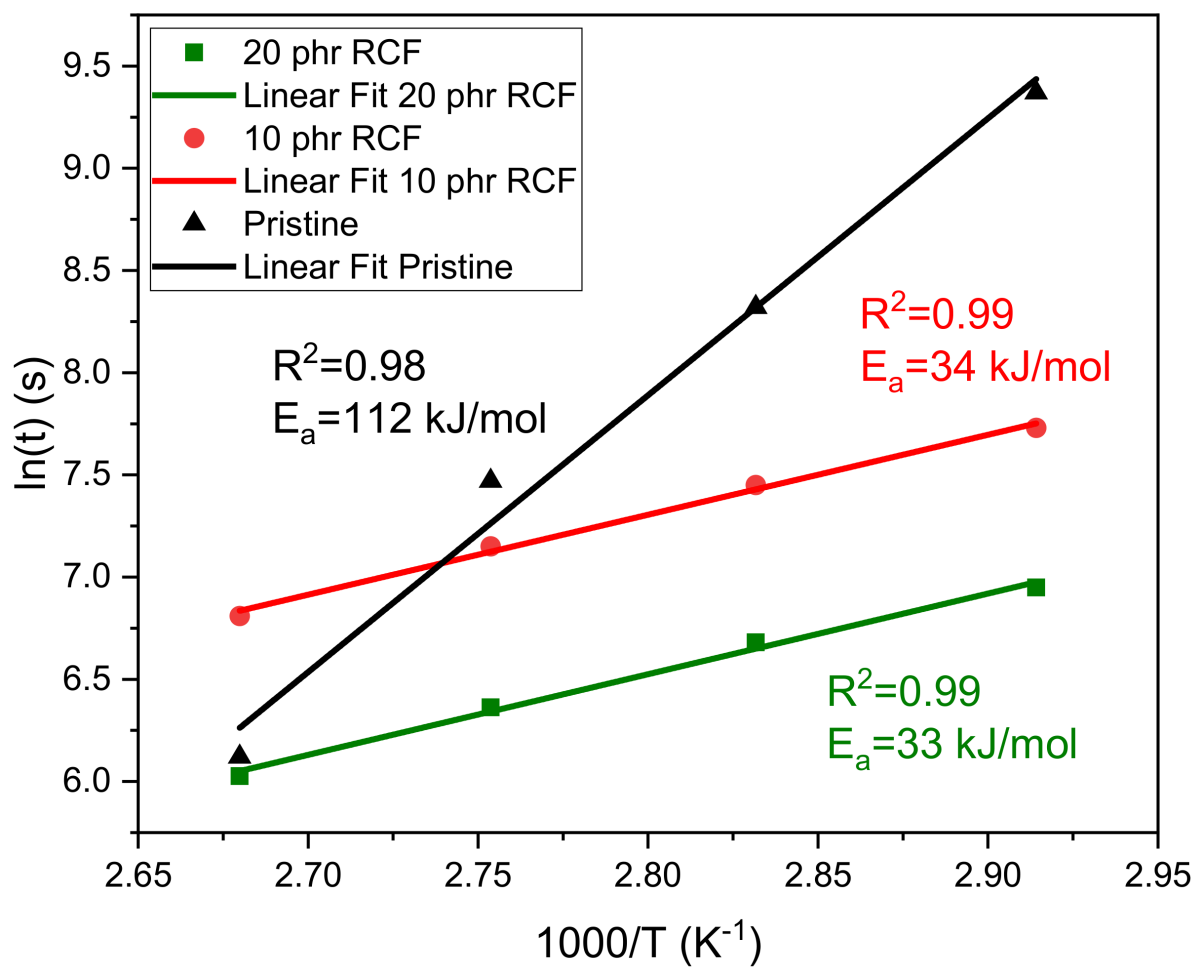
Arrhenius plot for all the studied samples.

The Arrhenius plot reported in Figure 12 exhibits a high degree of linear correlation (R^2^ ≥ 0.97–0.99), confirming the Arrhenius-type temperature dependence of the viscosity for all tested samples. The bond dynamics are therefore thermally activated, and the thermal response is governed by a well-defined energy barrier. The activation energies (Ea), derived from the slope of each linear fit, were calculated to be 112 kJ mol^−1^ for the unfilled system, and significantly lower values of 34 kJ mol^−1^ and 33 kJ mol^−1^ for the 10 phr and 20 phr RCF-containing formulations, respectively. These results confirm that the addition of recycled carbon fibres facilitates the dynamic exchange process. We suppose that the acceleration of stress relaxation with increasing RCF loading can be explained by considering different factors. First, the fibres emerge from the recycling process with surfaces rich in hydroxyl, carbonyl, and carboxyl groups; these sites act as Brønsted- or Lewis-acid centres that catalyze the associative ester-alcohol exchange, directly lowering the intrinsic activation barrier of the transesterification step. Second, once the RCF content approaches percolation, the graphitic network improves heat distribution throughout the sample, so a more uniform temperature is reached during testing, which registers as a smaller apparent activation energy. Third, fibre interfaces disrupt the epoxy network, generating a free volume that facilitates bond rearrangement. Fourth, the overall epoxy conversion after curing is slightly reduced as the filler fraction rises, which leaves a lower crosslink density and increases chain dynamics and reduces the energy cost of bond exchange.

These characterizations clearly show the dynamic covalent network properties of the crosslinked ECO-based coatings, which are not simply maintained in the presence of the carbon fibre filler but are enhanced.

### 3.6. Thermo-Electrical Measurements

Electrical conductivity was measured on crosslinked coatings using Ohm’s law. The slope of the I–V curve was used to determine the resistance, and the resistivity was then calculated. The conductivity was obtained as the inverse of the resistivity.

**Figure 13:**
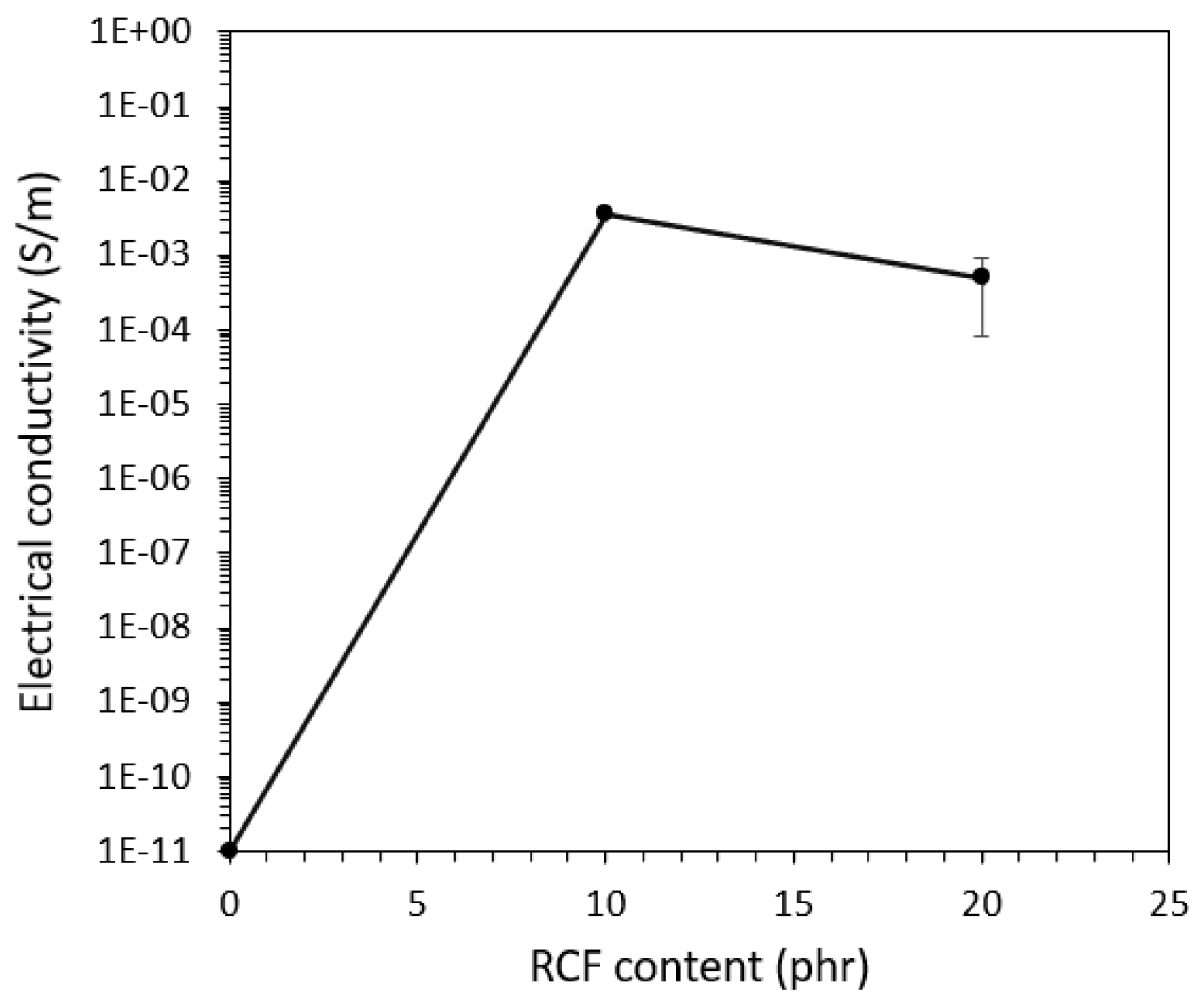
Electrical conductivity values as a function of RCF content.

The electrical conductivity values as a function of RCF content are presented in Figure 13, while the corresponding numerical data are summarized in Table 4. The incorporation of 10 phr RCF resulted in a significant increase in electrical conductivity compared with the crosslinked pristine coating. There is an increase in electrical conductivity from 1.00 × 10^−11^ S/m for the pristine UV-cured formulation (theoretical value) to 3.57 × 10^−3^ S/m for the crosslinked formulation containing 10 phr RCF. However, an unexpected decrease in conductivity to a value of 5.07 × 10^−4^ S/m was observed for the crosslinked composite containing 20 phr RCF, with an increase in the scattering of the results.

**Figure 14:**
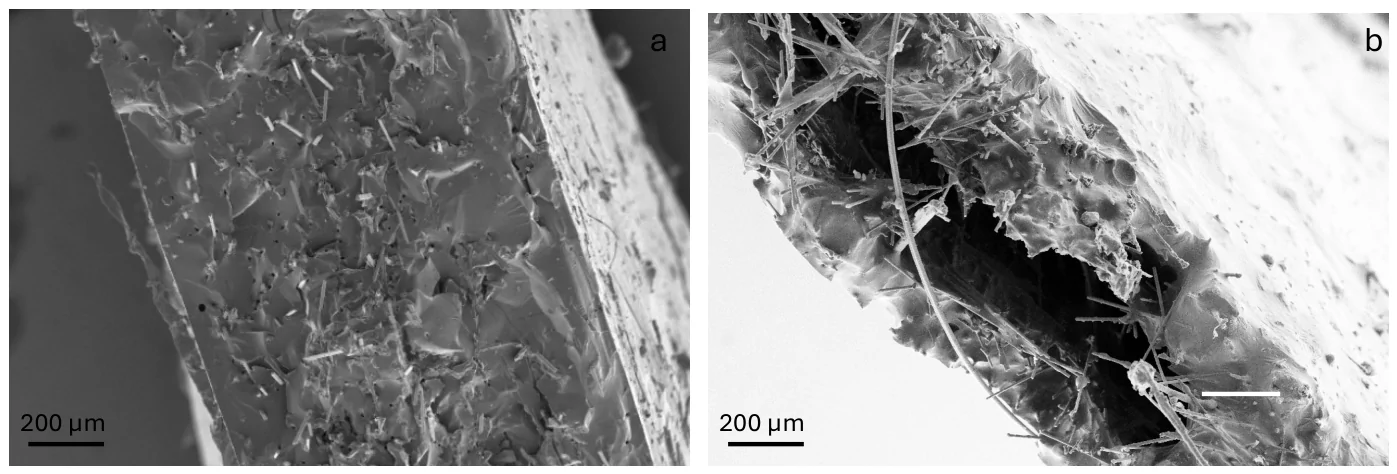
Magnifications of 10 phr RCF (**a**) and 20 phr RCF (**b**) cross sections.

To investigate this behaviour, the morphology of the composite coatings was examined by FESEM analysis. Cross-sectional FESEM micrographs are shown in Figure 14. The images reveal that increasing the RCF content from 10 to 20 phr promotes the formation of agglomerates and voids, leading to a less homogeneous filler distribution. This morphology hinders the formation of an effective conductive network, thereby slightly reducing the electrical percolation and, consequently, the electrical conductivity of the composite.

Following the electrical conductivity analysis, the Joule heating behaviour was investigated using the experimental setup described in Section 2.4.5. The measured values were consistent with the electrical conductivity observed at the corresponding RCF contents. For the 20 phr crosslinked coating, it was not possible to detect any heating effect due to the lower electrical conductivity, which led to electrical breakdown before any significant Joule heating could be established. Consequently, Joule heating data were collected only for the 10 phr sample.

**Figure 15:**
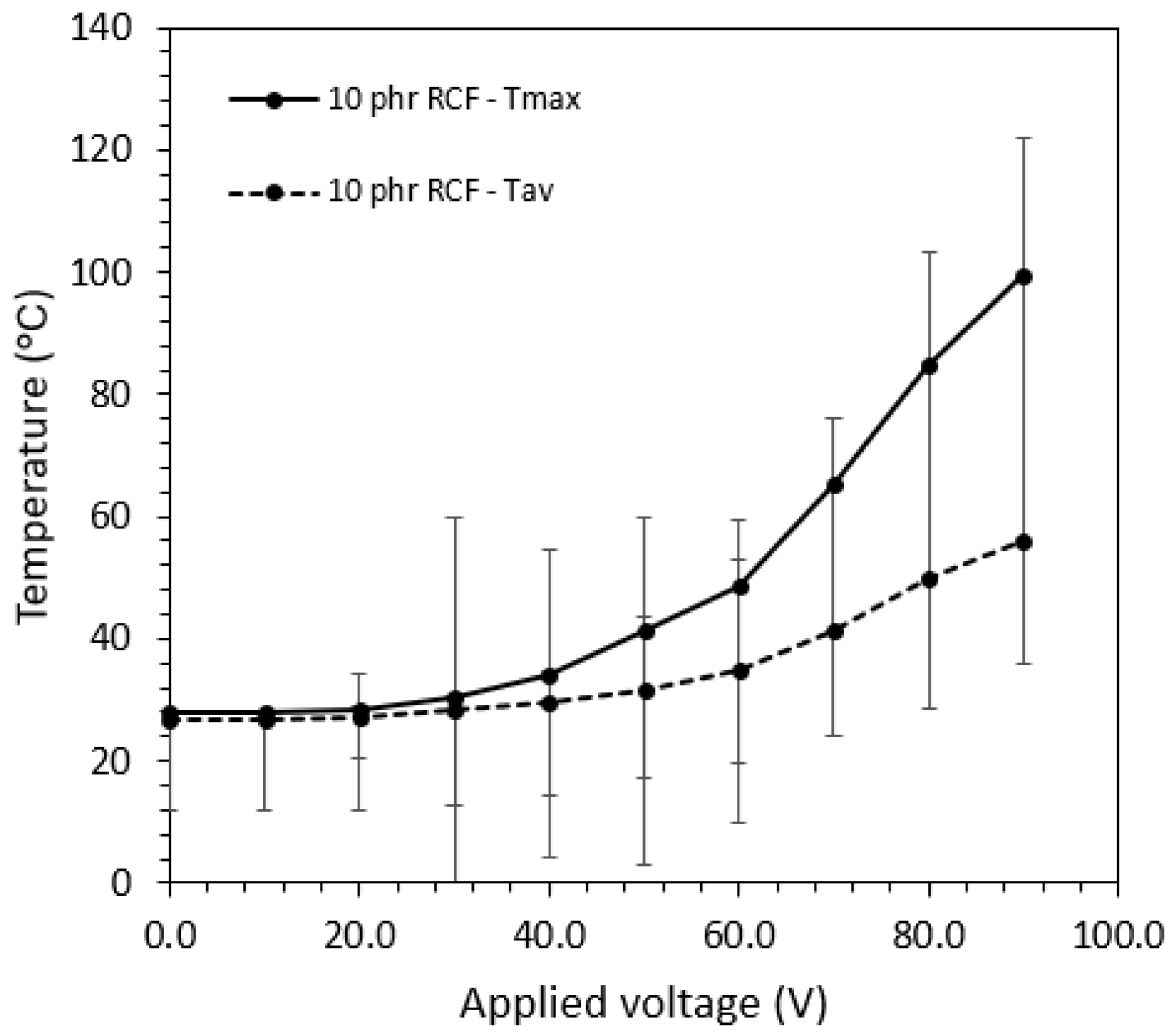
Temperature as a function of applied voltage.

Thet temperature profile as a function of applied voltage is reported in Figure 15 for the crosslinked coating containing 10 phr RCF.

**Figure 16:**
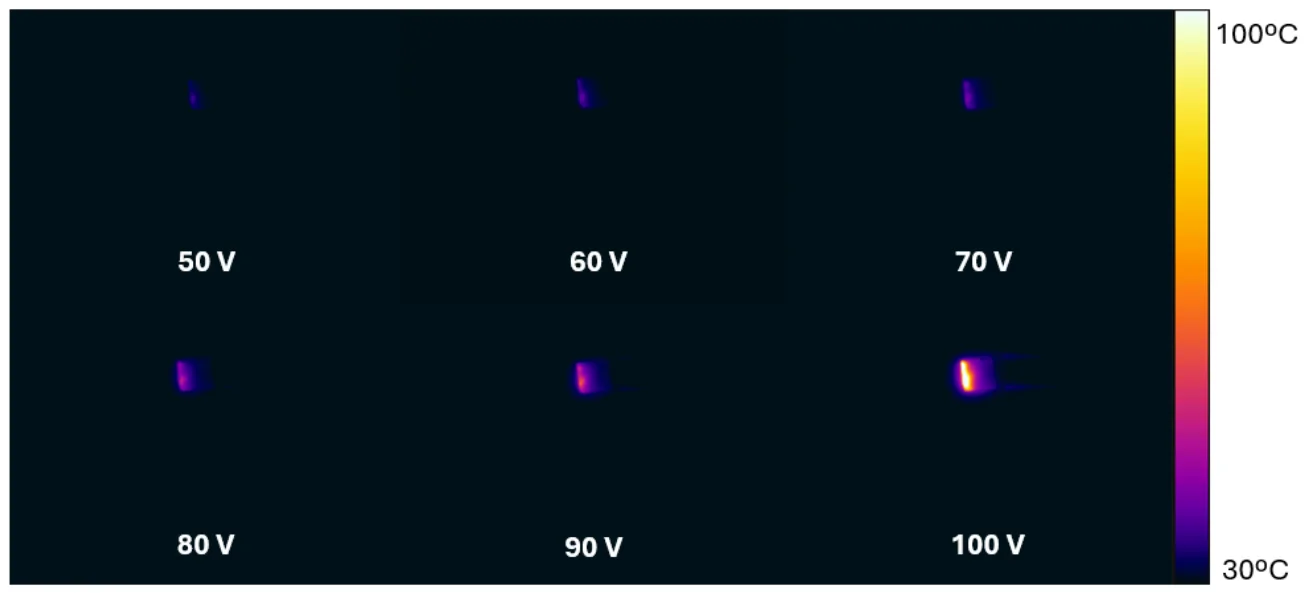
Thermographs of 10 phr RCF samples at different applied voltages.

In addition, it was possible to observe the heating behaviour through a thermal camera. However, as visible from Figure 16, the temperature distribution was not homogeneous, as the highest temperature was localized only in a restricted region of the specimen, for all the applied voltages, from 50 to 100 V. As we will report below, the temperature reached by the Joule effect could be sufficient to induce healing in damaged samples (see below); therefore, we could exploit the electrical conductivity achieved by dispersing the RCF to induce healing with the Joule effect by simply applying voltage to the damaged samples.

### 3.7. Healing Evaluation

A non-conventional macroscopic approach was adopted to evaluate the healing behaviour of the material. Micro-tensile tests were performed using the experimental setup described in Section 2.4.7 to assess the mechanical performance of the repaired specimens.

Two healing conditions were investigated. In the first, the overlapped specimens were treated at 70 °C for 30 min. In the second, the specimens underwent a healing treatment at 120 °C for 6 h. These temperatures were selected since the feasibility of reaching them using the Joule-heating effect was demonstrated by applying voltage.

The maximum tensile load sustained by each healed specimen was measured after thermal healing. The results are reported in Table 5 as the maximum load (N) supported by specimens with identical geometry and are compared with the tensile performance of the reference specimens.

The sample containing 10 phr RCF, when subjected to the first healing condition (70 °C for 30 min) for the micro-tensile test, failed in the non-overlapping region at slightly lower forces compared to the reference samples (see data reported in Table 5). This behaviour is likely due to a slightly reduced thickness in that section and the partial misalignment in the overlapped samples, suggesting that the healing process was successful.

When the sample containing 20 phr was subjected to the first healing conditions, a detachment of the upper part of the specimen was observed after the micro-tensile test. This is likely an indication that the fibre content and the selected time–temperature combination were not sufficient to achieve optimal healing.

For the second healing condition (120 °C for 6 h), both 10 phr and 20 phr sample failures occurred near the joint during tensile testing, but at higher force levels. This could be explained considering the occurrence of thermal post-curing of the samples, increasing the conversion and therefore the performance of the materials. Overall, this last healing condition was largely efficient, permitting the creation of perfectly healed joints.

The lower values of tensile load obtained by increasing the filler load in all the cases are potentially due to a lack of matrix conversion and the presence of voids in the bulk of the samples, as already explained.

## 4. Conclusions

This work demonstrates that combining a bio-based dynamic matrix (ECO/DBP) with recycled carbon fibres is a viable strategy for producing sustainable, multifunctional, healable coatings. The main findings can be summarized as follows.

The incorporation of RCF partially hindered UV curing, lowering both the conversion and photopolymerization rate as filler content increased, most likely due to competitive UV absorption by the fibres and reduced mobility of reactive species within the percolated network. Despite this, gel content remained above 98% for all formulations, confirming that crosslinking efficiency was not compromised.

Thermo-mechanical analysis showed that RCF addition did not significantly affect the glass transition temperature of crosslinked coatings but substantially increased the rubbery storage modulus and the apparent crosslink density, an effect ascribed to physical reinforcement and interfacial chain immobilization rather than additional chemical crosslinking, and a distinction supported by the unchanged conversion values from FT-IR and DSC.

Stress relaxation experiments confirmed that all crosslinked formulations retain dynamic characteristics, with Arrhenius behaviour and well-defined activation energies. Notably, the presence of RCF lowered the activation energy for bond exchange by roughly a factor of three compared to the pristine crosslinked formulation, suggesting that residual functional groups and trace metal species on the recycled fibre surface actively participate in catalyzing the transesterification network, in addition to effects linked to heat distribution and reduced crosslink density at a higher filler content.

Thermo-electrical characterization showed that the UV-cured formulation containing 10 phr RCF evidenced an important enhancement of electrical conductivity as well as temperature enhancement due to the Joule effect.

In fact, the 10 phr RCF crosslinked formulation offered the best compromise between processability and performance: it reached the highest electrical conductivity among the composites and was the only formulation in which controlled Joule heating could be reliably measured, whereas the 20 phr system experienced voids and lower conversion, which suppressed both conductivity and the heating response.

Finally, thermally activated healing was confirmed for both RCF loadings, with the most severe condition (120 °C, 6 h) restoring tensile strength and permitting higher values than that of the undamaged reference specimens, while the milder condition (70 °C, 30 min) produced only partial recovery, particularly for material containing higher filler content. Taken together, these results indicate that filler loading must be carefully optimized, since excessive RCF content, while beneficial for mechanical reinforcement, can compromise curing efficiency, electrical percolation, and healing performance. Overall, the ECO/DBP/RCF system developed here represents a promising route toward recyclable, electrically responsive, and repairable coatings derived largely from renewable and reclaimed materials, with the 10 phr formulation identified as the most balanced candidate for further development.

## Figures and Tables

**Table 1:** Components for each formulation.

Formulation	RCF (phr)	RCF (%wt)	PI (phr)	ITX (phr)	DBP (phr)
Pristine	-	-	2	2	15
10 phr RCF	10	9.09			
20 phr RCF	20	16.67			

**Table 2:** FTIR and p-DSC conversions, maximum R_p_ and gel content values.

Formulation	Conversion _FTIR_ (%)	Conversion _DSC_ (%)	Maximum of R_p_ (s^−1^)	Gel Content (%)
Pristine	82 ± 3	77 ± 1	0.025 ± 0.001	99.9 ± 0.1
10 phr RCF	77 ± 1	74 ± 1	0.023 ± 0.001	99.8 ± 0.1
20 phr RCF	74 ± 2	61 ± 3	0.020 ± 0.03	98.2 ± 0.1

**Table 3:** T_g_ values registered through DMTA, DSC and ν_c,app._ trends as a function of RCF amount.

Formulation	T_g, DMTA_ (°C)	T_g, DSC_ (°C)	ν_c,app._ (mol/m^3^)
Pristine	2 ± 1	−18 ± 1	7 ± 1
10 phr RCF	0 ± 1	−18 ± 3	298 ± 9
20 phr RCF	2 ± 1	−17 ± 2	320 ± 12

**Table 4:** Average electrical conductivity values at different RCF loads, with standard deviation.

RCF Content (phr)	Electrical Conductivity (S/m)	Stdev
0	1.00 × 10^−11^	0 (theoretical value)
10	3.57 × 10^−3^	9.89 × 10^−5^
20	5.07 × 10^−4^	4.24 × 10^−4^

**Table 5:** Maximum tensile loads of 10 and 20 phr RCF of reference specimens under different healing conditions.

**Reference**	**Tensile Load [N]**	
10 phr RCF	0.51 ± 0.25	
20 phr RCF	0.25 ± 0.10	
Partial overlap	**First condition** 70 °C—30 min	**Second condition** 120 °C—6 **h**
10 phr RCF	0.40 ± 0.26	1.33 ± 0.70
20 phr RCF	0.25 ± 0.04	1.04 ± 0.40

## Data Availability

The original contributions presented in this study are included in the article. Further inquiries can be directed to the corresponding author(s).

## References

[1] Alarcon R.T., Cellai A., Porcarello M., Sölle B., Rossegger E., Schmitt C.C., Sangermano M. (2025). Green Design of Renewable Dual-Curing Polymers with Self-Healing and Recyclable Networks for 3D Printing. ACS Sustain. Chem. Eng..

[2] Fortman D.J., Brutman J.P., De Hoe G.X., Snyder R.L., Dichtel W.R., Hillmyer M.A. (2018). Approaches to Sustainable and Continually Recyclable Cross-Linked Polymers. ACS Sustain. Chem. Eng..

[3] Bachmann J., Yi X., Tserpes K., Sguazzo C., Barbu L.G., Tse B., Soutis C., Ramón E., Linuesa H., Bechtel S. (2021). Towards a Circular Economy in the Aviation Sector Using Eco-Composites for Interior and Secondary Structures. Results and Recommendations from the EU/China Project ECO-COMPASS. Aerospace.

[4] Tserpes K., Tzatzadakis V. (2022). Life-Cycle Analysis and Evaluation of Mechanical Properties of a Bio-Based Structural Adhesive. Aerospace.

[5] Righetti G.I.C., Faedi F., Famulari A. (2024). Embracing Sustainability: The World of Bio-Based Polymers in a Mini Review. Polymers.

[6] Rahman M.Z., Rahman M., Mahbub T., Ashiquzzaman M., Sagadevan S., Hoque M.E. (2023). Advanced Biopolymers for Automobile and Aviation Engineering Applications. J. Polym. Res..

[7] Rathi S., Saka R., Domb A.J., Khan W. (2019). Protein-Based Bioadhesives and Bioglues. Polym. Adv. Technol..

[8] Sangermano M., Bergoglio M., Schögl S. (2024). Biobased Vitrimeric Epoxy Networks. Macromol. Mater. Eng..

[9] Law K.L., Starr N., Siegler T.R., Jambeck J.R., Mallos N.J., Leonard G.H. (2020). The United States’ Contribution of Plastic Waste to Land and Ocean. Sci. Adv..

[10] Mendes-Felipe C., Oliveira J., Etxebarria I., Vilas-Vilela J.L., Lanceros-Mendez S. (2019). State-of-the-Art and Future Challenges of UV Curable Polymer-Based Smart Materials for Printing Technologies. Adv. Mater. Technol..

[11] Javier F., Orús E., Farrés Q.T., Dagdag O., Kim H. (2024). Recent Advances in Fire Safety of Carbon Fiber-Reinforced Epoxy Composites for High-Pressure Hydrogen Storage Tanks. Polymers.

[12] Ali Z., Yaqoob S., Yu J., D’Amore A., Fakhar-e-Alam M., Ali Z., Yaqoob S., Yu J., D’Amore A., Fakhar-e-Alam M. (2024). A Comparative Review of Processing Methods for Graphene-Based Hybrid Filler Polymer Composites and Enhanced Mechanical, Thermal, and Electrical Properties. J. King Saud Univ. Sci..

[13] Caminero M.A., Pavlopoulou S., Lopez-Pedrosa M., Nicolaisson B.G., Pinna C., Soutis C. (2013). Analysis of Adhesively Bonded Repairs in Composites: Damage Detection and Prognosis. Compos. Struct..

[14] Sekine H., Fujimoto S.E., Okabe T., Takeda N., Yokobori T. (2006). Structural Health Monitoring of Cracked Aircraft Panels Repaired with Bonded Patches Using Fiber Bragg Grating Sensors. Appl. Compos. Mater..

[15] Castellazzi G., Krysl P., Bartoli I. (2013). A Displacement-Based Finite Element Formulation for the Analysis of Laminated Composite Plates. Compos. Struct..

[16] Heaney M.B. (1996). Resistance-expansion-temperature Behavior of a Disordered Conductor–Insulator Composite. Appl. Phys. Lett..

[17] Scarisbrick R.M. (1973). Electrically Conducting Mixtures. J. Phys. D Appl. Phys..

[18] Zhang W., Dehghani-Sanij A.A., Blackburn R.S. (2007). Carbon Based Conductive Polymer Composites. J. Mater. Sci..

[19] Espeute E., Martinez-Diaz D., Vázquez Sánchez P., Martín Z., Del Rosario G., Jiménez-Suárez A., Prolongo S.G. (2024). Smart Electroactive Self-Repairable Coating Involving End-of-Life Aircraft Prepregs by Mechanical Recycling. J. Clean. Prod..

[20] Sau K.P., Chaki T.K., Khastgir D. (1997). Conductive Rubber Composites from Different Blends of Ethylene-Propylene-Diene Rubber and Nitrile Rubber. J. Mater. Sci..

[21] Martinez-Diaz D., Espeute E., Jiménez-Suárez A., Prolongo S.G. (2023). Electrical and Joule Heating Capabilities of Multifunctional Coatings Based on Recycled Carbon Fiber from Prepreg Scrap. ACS Omega.

[22] Moraru D., Cortés A., Martinez-Diaz D., Prolongo S.G., Jiménez-Suárez A., Sangermano M. (2024). Sustainable Electrically Conductive Bio-Based Composites via Radical-Induced Cationic Frontal Photopolymerization. Polymers.

[23] Cellai A., Pezzana L., Matsukawa K., Jiménez-Suárez A., Sangermano M., Cortés A. (2026). Vat Photopolymerization of Bio-Derived Conductive Composites for Smart Devices with Joule Heating Capabilities. Prog. Addit. Manuf..

[24] Alarcon R.T., Lamb K.J., Bannach G., North M. (2021). Opportunities for the Use of Brazilian Biomass to Produce Renewable Chemicals and Materials. ChemSusChem.

[25] Pezzana L., Wolff R., Stampfl J., Liska R., Sangermano M. (2024). High Temperature Vat Photopolymerization 3D Printing of Fully Bio-Based Composites: Green Vegetable Oil Epoxy Matrix & Bio-Derived Filler Powder. Addit. Manuf..

[26] Jha S., Akula B., Enyioma H., Novak M., Amin V., Liang H. (2024). Biodegradable Biobased Polymers: A Review of the State of the Art, Challenges, and Future Directions. Polymers.

[27] Velasquez S.T.R., Hu Q., Kramm J., Santin V.C., Völker C., Wurm F.R. (2025). Plastics of the Future? An Interdisciplinary Review on Biobased and Biodegradable Polymers: Progress in Chemistry, Societal Views, and Environmental Implications. Angew. Chem. Int. Ed..

[28] Porcarello M., Greco E., Cellai A., Turra Alarcon R., Rossegger E., Sangermano M. (2026). 3D Printing with Biobased Epoxidized Formulations Based on Vegetable Oils with Dynamic Polymer Network Properties. Polym. Chem..

[29] Yao H., Xu X., Chen Z., Li K., Wang T., Pei S., Fu J., Wang J. (2025). A Review of Epoxy Vitrimer-Based Thermally Conductive Composites. RSC Adv..

[30] Pezzana L., Malmström E., Johansson M., Sangermano M. (2021). Uv-Curable Bio-Based Polymers Derived from Industrial Pulp and Paper Processes. Polymers.

[31] Denissen W., Winne J.M., Du Prez F.E. (2016). Vitrimers: Permanent Organic Networks with Glass-like Fluidity. Chem. Sci..

[32] Guggari S., Magliozzi F., Malburet S., Graillot A., Destarac M., Guerre M. (2023). Vanillin-Based Epoxy Vitrimers: Looking at the Cystamine Hardener from a Different Perspective. ACS Sustain. Chem. Eng..

[33] Genua A., Montes S., Azcune I., Rekondo A., Malburet S., Daydé-Cazals B., Graillot A. (2020). Build-to-Specification Vanillin and Phloroglucinol Derived Biobased Epoxy-Amine Vitrimers. Polymers.

[34] Zhang Y.-H., Zhai M.-J., Shi L., Lei Q.-Y., Zhang S.-T., Zhang L., Lyu B., Zhao S.-H., Ma J.-Z., Thakur V.K. (2023). Sustainable Castor Oil-Based Vitrimers: Towards New Materials with Reprocessability, Self-Healing, Degradable and UV-Blocking Characteristics. Ind. Crops Prod..

[35] Cellai A., Alarcon R.T., Sölle B., Rossegger E., Casalegno V., Salvo M., Sangermano M. (2025). Biobased Epoxidized Castor Oil Covalent Adaptable Networks Adhesives Reinforced with Recycled Carbon Fibers. Macromol. Rapid Commun..

[36] Alarcon R.T., Gaglieri C., Lamb K.J., North M., Bannach G. (2020). Spectroscopic Characterization and Thermal Behavior of Baru Nut and Macaw Palm Vegetable Oils and Their Epoxidized Derivatives. Ind. Crops Prod..

[37] Smith B.C. (2022). The Infrared Spectra of Polymers V: Epoxies. Spectroscopy.

[38] Pandita S.D., Wang L., Mahendran R.S., MacHavaram V.R., Irfan M.S., Harris D., Fernando G.F. (2012). Simultaneous DSC-FTIR Spectroscopy: Comparison of Cross-Linking Kinetics of an Epoxy/Amine Resin System. Thermochim. Acta.

[39] González M.G., Cabanelas J.C., Baselga J. (2012). Applications of FTIR on Epoxy Resins—Identification, Monitoring the Curing Process, Phase Separation and Water Uptake. Infrared Spectrosc.-Mater. Sci. Eng. Technol..

[40] Liu X., Yang X., Wang S., Wang S., Wang Z., Liu S., Xu X., Liu H., Song Z. (2021). Fully Bio-Based Polyhydroxyurethanes with a Dynamic Network from a Terpene Derivative and Cyclic Carbonate Functional Soybean Oil. ACS Sustain. Chem. Eng..

[41] Mądry K., Nowicki W. (2021). Wetting between Cassie–Baxter and Wenzel Regimes: A Cellular Model Approach. Eur. Phys. J. E.

[42] Hejazi V., Moghadam A.D., Rohatgi P., Nosonovsky M. (2014). Beyond Wenzel and Cassie–Baxter: Second-Order Effects on the Wetting of Rough Surfaces. Langmuir.

[43] Vigdorowitsch M., Tsygankova L.E., Ostrikov V.V., Rodionova L.D. (2022). Beyond the Wenzel and Cassie–Baxter World: Mathematical Insight into Contact Angles. Math. Methods Appl. Sci..

[44] Tobolsky A.V. (1956). Stress Relaxation Studies of the Viscoelastic Properties of Polymers. J. Appl. Phys..

[45] Brutman J.P., Fortman D.J., De Hoe G.X., Dichtel W.R., Hillmyer M.A. (2019). Mechanistic Study of Stress Relaxation in Urethane-Containing Polymer Networks. J. Phys. Chem. B.

[46] Obaid N., Kortschot M.T., Sain M. (2017). Understanding the Stress Relaxation Behavior of Polymers Reinforced with Short Elastic Fibers. Materials.

